# Prenatal anxiety, breastfeeding and child growth and puberty: linking evolutionary models with human cohort studies

**DOI:** 10.1080/03014460.2020.1751286

**Published:** 2020-05-20

**Authors:** Sinead English, India Wright, Verity Ashburn, Gemma Ford, Doretta Caramaschi

**Affiliations:** a School of Biological Sciences, University of Bristol, Bristol, UK;; b Reproduction and Development Programme, Bristol Medical School (Translational Health Sciences), University of Bristol, Bristol, UK;; c Medical Research Council Integrative Epidemiology Unit, Bristol Medical School (Population Health Sciences), University of Bristol, Bristol, UK

**Keywords:** Stress, lactation, breastfeeding, ALSPAC, cohort study

## Abstract

**Background:** Stress experienced by mothers during pregnancy can have both immediate and long-term effects on child development, potentially mediated by breastfeeding.

**Aim:** Using a UK birth cohort study, we asked how maternal stress relates to breastfeeding and consequences for growth and puberty onset.

**Subjects and methods:** We analysed data from the Avon Longitudinal Study of Parents and Children, collected via questionnaires and clinic visits (N: 698–8,506). We used reports of prenatal anxiety, breastfeeding, early growth and age at menarche or first voice change. Confounding by maternal age, parity, smoking, education and body mass index (BMI) was considered.

**Results:** Mothers with higher levels of reported anxiety were less likely to breastfeed (Odds ratio (OR): 0.83, 95% confidence interval (CI): 0.71, 0.97). Breastfed infants had slower growth before weaning, although growth differences were unclear thereafter. Being breastfed for more than six months was associated with later puberty onset in females (2.76 months later than non-breastfed; CI: 0.9, 4.63), although the association was attenuated by confounders and BMI (1.51 months, CI: −0.38, 3.40). No association between breastfeeding and puberty onset in males was found.

**Conclusion:** Our studies fit results shown previously, and we consider these in light of evolutionary life history theory while discussing key challenges in such an approach.

## Introduction

A growing body of studies on human and non-human animals have shown that maternal stress experienced during gestation has both immediate physiological effects on the development of offspring *in utero* (Kinsella and Monk 2009), as well as longer term consequences for later health, behaviour and life history (Entringer et al. 2012; Sheriff and Love 2013; Aizer et al. 2016). Some of these longer-term effects may be explained by the relationship between maternal stress and breastfeeding, as stressed mothers tend to be less likely to initiate breastfeeding or breastfeed their children as long (Li et al. 2008; Ystrom 2012; Dozier et al. 2012), with similar associations between elevated physiological stress and reduced lactation found in animal studies (Lau 2001). Note that the term ‘stress’ can refer to direct physiological measures – such as circulating cortisol levels – or to the experience of stressful events such as predators (Sheriff and Love 2013) or natural disasters (Duchesne et al. 2017), and may also be reflected, in humans, in measures of self-reported anxiety (Nawa et al. 2019).

**Figure 1.**
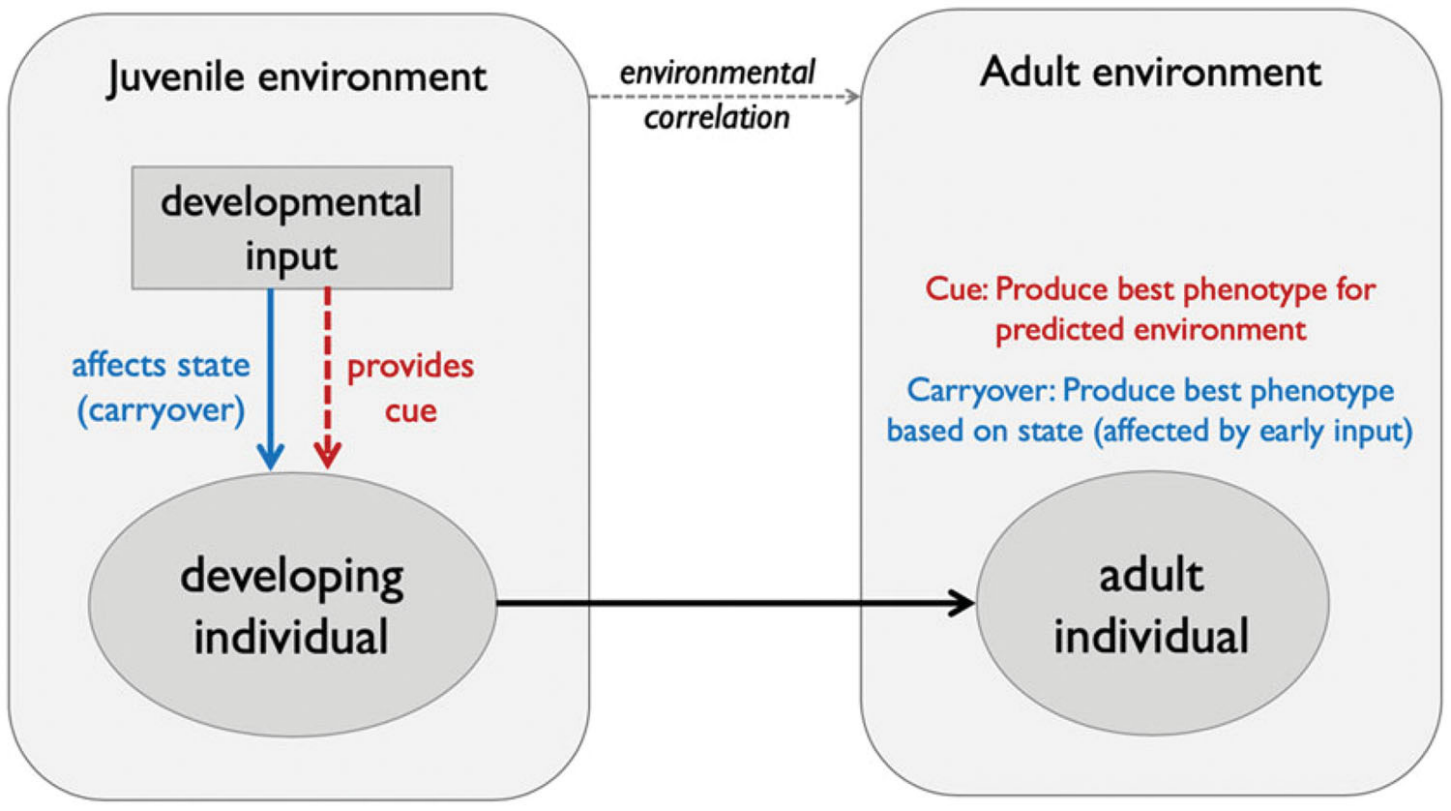
Schematic showing the link between the juvenile environment (e.g. exposed to maternal stress or not) and development outcomes for offspring when maternal stress provides a cue of later offspring conditions (predictive adaptive response depending on environmental correlation, red text) and when maternal stress imposes a negative cost on offspring development (developmental constraint due to a carryover effect of early input, blue text).

From an evolutionary life history perspective, several explanations have been proposed for the association between prenatal stress and child outcomes. These fall broadly under two categories: developmental constraints imposed by early stress, or adaptive plasticity in response to cues of environmental adversity (Figure 1, see Monaghan 2008; Nettle et al. 2013; Uller et al. 2013; Nettle and Bateson 2015 for general discussion, and Sheriff and Love 2013; Berghänel et al. 2017 on stress). The developmental constraint argument is that long-term effects of exposure to maternal stress are non-adaptive. Stressed mothers have less resources at their disposal to invest in young, thus producing smaller offspring who incur a fitness disadvantage due to this maternal stress. In contrast, the adaptive plasticity explanation proposes that stressed mothers induce the development of adaptations in their offspring that lead to a phenotype adapted to a stressful world (often termed a ‘predictive adaptive response’). More specifically, offspring may follow an accelerated life strategy (Dammhahn et al. 2018) as an adaptation to developing in a harsh environment, with rapid growth and earlier age at reproductive maturity to ensure reproductive success in the face of lower expected lifespan (Gluckman and Hanson 2006). This argument relies on the maternal environment being predictive of later offspring conditions, which can be problematic in species like humans which live in variable environments and have relatively longer lifespans (Wells 2012).

These two explanations are not mutually exclusive, and it is possible that in many systems both processes are at play. In the context of maternal stress, Berghänel et al. (2017) have recently proposed an integrative framework to combine both constraint and adaptive plasticity responses. They suggest that developmental constraints and adaptive plasticity can act in opposing ways: stressed mothers produce smaller offspring with lower intrauterine growth, but these offspring then exhibit rapid postnatal growth, thus counter-acting the earlier maternal constraint. They test their predictions using comparative data across 21 non-human mammal species and find that maternal stress does indeed restrict pre-weaning offspring growth but, particularly when stress is measured early in gestation, offspring follow more rapid growth thereafter (Berghänel et al. 2017). How these effects might play out in the context of humans, and may be mediated by the process of breastfeeding, remains an open question. It is very challenging to consider these evolutionary processes in the context of human breastfeeding, both because breastfeeding outcomes are determined not only by maternal stress but by a suite of social, economic and cultural factors; and because babies who are not breastfed are supplemented by formula milk.

Several studies have reported associations between prenatal stress and later growth in childhood and age at reproductive maturity. Many of these have focussed on prenatal anxiety self-reported by mothers, while others have used quasi-experimental data by following children born to mothers who experienced natural disasters during pregnancy. A recent study using the same cohort from the present analysis measured the association between maternal anxiety during pregnancy and child growth, and found slightly higher growth between 25–31 months of age in those born to anxious mothers (Nawa et al. 2019). These effects on growth can have long-term consequences: a separate study, following children born to mothers who experienced stress during a natural disaster in the form of Quebec’s January 1998 ice storm during pregnancy, found that this prenatal anxiety was associated with earlier age at menarche, mediated through effects on body mass index at 5.5 years (Duchesne et al. 2017). It is currently unclear to what extent the association between prenatal anxiety and breastfeeding might mediate these interactions between prenatal anxiety and later growth and age at onset of puberty. Such an effect is quite possible, as several studies already demonstrate a negative association between elevated maternal stress and breastfeeding, although these have small sample sizes, recall bias due to the use of retrospective questionnaires, or inappropriate adjustment for confounding (Dozier et al. 2012; Fallon et al. 2016; Buck et al. 2018; Bublitz et al. 2019).

Here, we build on this earlier work and ask – using data from a large, prospective study, which allows consideration of several confounding variables to account for relevant aspects of maternal environment and context – whether maternal prenatal anxiety is associated with later child growth and onset of reproductive maturity, and if this is mediated through breastfeeding. More precisely, we ask the following questions:

1. Is maternal stress, as measured by self-reported anxiety, associated with breastfeeding?
2. Are there differences in growth of children according to breastfeeding experience and/or maternal anxiety, and does this depend on the stage at which growth is measured (before versus after weaning)?
3. What is the association between maternal anxiety and/or breastfeeding experience and the age of puberty onset?

We predicted that, as shown in previous work (see above), mothers with higher anxiety will breastfeed less, and that their children will have more rapid growth and earlier onset of puberty. We tested whether any associations between prenatal anxiety and later growth/puberty outcomes are mediated through breastfeeding and growth. While the effects of breastfeeding on growth and puberty have received considerable attention, and some studies have investigated effects of prenatal anxiety on growth and puberty, our study is one of the first to combine these all together and consider explicitly the role of breastfeeding, and the possible fit of expectations drawn from evolutionary life history theory. We also consider the limitations and caveats of making such links, particularly in our contemporary dataset where supplementation or replacement of breastfeeding with formula milk is an important factor to consider and potentially weakens the parallels to be drawn with non-human mammal studies.

## Subjects and methods

We asked the above questions analysing data from the Avon Longitudinal Study of Parents and Children (ALSPAC). This is an extensive population study aimed at examining the role of genes and environment in shaping health and development in parents and children (Golding et al. 2001; Boyd et al. 2013; Fraser et al. 2013). A total of 14,541 pregnant women in the Bristol area were recruited into the study during the period 1990–1992. Our analyses considered a subset of these pregnancies, with specific sample sizes provided in the sections below (ranging from 698 on the growth subset, to 8,506 on breastfeeding outcome). Ethical approval for the study was obtained from the ALSPAC Ethics and Law Committee and the Local Research Ethics Committees. Informed consent for the use of data collected via questionnaires and clinics was obtained from participants following the recommendations of the ALSPAC Ethics and Law Committee at the time. Further details on the study methodology are available at the ALSPAC website (http://www.alspac.bris.ac.uk). Please note that the study website contains details of all the data that is available through a fully searchable data dictionary and variable search tool (http://www.bristol.ac.uk/alspac/researchers/our-data/). Below, we describe specific methodology related to our research questions.

### What is the relationship between prenatal anxiety and breastfeeding?

To investigate the link between prenatal anxiety and breastfeeding, we included variables from questionnaires completed by women at different stages of pregnancy and post-partum. In total, complete records were available for 8,506 women for these analyses.

#### Anxiety

We used self-reported levels of anxiety as our measure of prenatal stress, as direct physiological measures were not available, and this anxiety measure has been used to link maternal stress and child behaviour in other studies (Hines et al. 2002; O’Connor et al. 2002). Reported anxiety scores were derived from structured questionnaire responses at 32 weeks’ gestation and transformed into the Crown-Crisp Experiential Index (CCEI). Previous studies have shown that the CCEI responses correlate with both state- and trait-based aspects of the more commonly used Spielberger State-Trait Anxiety Inventory (Heron et al. 2004; Leis et al. 2014), and thus reflect generalised anxiety as well as feelings specific to being pregnant, rather than concerning anxiety over the future health of the child. The initial CCEI scale ranges from 1 to 16, but for ease of analysis, we transformed anxiety to a four-factor scale according to quartiles of the data: (i) no anxiety (CCEI score 0–2); (ii) mild anxiety (3–4); (iii) moderate anxiety (5–7); and (iv) severe anxiety (8–16), see O’Connor et al. (2002); Cookson et al. (2009) for a similar approach. To check the robustness of our results to this categorisation, we also tested associations between traits of interest and anxiety measured on a continuous scale (1–16), with the quartiles as linear, and using a cut-off of the top 15% (CCEI score 9 or above).

#### Breastfeeding

We included two measures for breastfeeding status, based on questionnaire responses when babies were 15 months of age. ‘Ever breastfed’ was scored as 1 for those mothers who reported some breastfeeding and 0 for those who recorded none. We also included data on the duration of breastfeeding, categorised as whether breastfeeding lasted for <6 or ≥6 months. We used 6 months as a cut-off as this is the time recommended by the World Health Organisation to breastfeed exclusively, as well as previous studies having shown that, beyond this point, only 33% of mothers are still breastfeeding in this population (Donath and Amir 2007).

#### Confounding variables

We included several confounding variables based on previous studies on the link between prenatal anxiety (Goyal et al. 2010; Biaggi et al. 2016) and breastfeeding outcomes (Meedya et al. 2010; Zhu et al. 2015; Wallwiener et al. 2016), as well as other studies in ALSPAC on factors affecting breastfeeding, growth and puberty onset (Ong et al. 2002; Ong et al. 2009): (i) Maternal education, whether a mother has a university degree (1) or not (0): (ii) Parity, previous child (1) or not (0); (iii) Maternal smoking, mother smoked at all during pregnancy (1) or not (0); (iv) Maternal body mass index pre-pregnancy.

### What is the association between breastfeeding and later growth and puberty onset?

We measured the association between breastfeeding and growth during early and later infancy, and the association between breastfeeding and the age at onset of puberty using the following descriptions.

#### Growth during infancy and childhood

Although there have been extensive studies investigating effects of breastfeeding on growth (e.g. Ong et al. 2002), we conducted our own analyses here on the link between breastfeeding, anxiety measures and other maternal factors on growth at different time-points of development, to relate our results to the comparative study by Berghänel et al. (2017). We used data on body mass attained during clinic visits for children included in the “Children In Focus” subset (∼10% of the cohort, Golding et al. 2001), who were assessed at ten intervals between 4 and 61 months for measures on growth amongst other factors.

The time-points we included here include early infancy growth (pre-weaning) (0 to 8 months), later infancy growth (8 to 25 months) and childhood (25 to 61 months). These time windows did not completely align with our breastfeeding intervals (e.g. 0–6 months) owing to the schedule of clinic visits in the data. The last time window covers a broad interval (∼3 years) but we selected this period to investigate early-childhood growth beyond the time of weaning, in line with other studies (Berghänel et al. 2017). We measured growth as the difference in body mass between the two time-points in question and controlled for mass at the start of the time-point in our analysis (see English et al. (2014) for a similar approach). For this analysis, we included breastfeeding categorised in such a way to account for both the initiation and duration of breastfeeding: Never breastfed, breastfed <6 months, and breastfed ≥6 months. We also considered all other confounding variables mentioned above, as well as child sex. Complete records on breastfeeding, growth and confounding variables were available for 945 children (0–8 months), 828 children (8–25 months) and 698 children (25–61 months).

#### Age at puberty onset

For girls, we noted the age at which the first period was recorded from any of the questionnaires between the 9-year and 17-year visit. When several different records were provided across the years for the same individual, we selected the minimum value. For boys, we recorded the first age-at-questionnaire when the respondent reported that his voice had started to change (Hollis et al. 2018). As absolute dates were not provided for this measure, we categorised the data as whether or not voice change had occurred before or after the median age at which voice change was reported (i.e., 14.6 years). In analyses of age at puberty onset, we considered BMI at 10 years as a possible mediator. BMI was calculated from the clinic records at the 10-year visit as an individual’s weight/height^2^ (in kg/m^2^). We selected the 10-year age-point as this was as close as possible to prepubertal BMI in our dataset, although menarche can occur before this age (Rogers et al. 2010). Complete records were available on breastfeeding, puberty onset and confounding variables for 2,281 girls and 1,936 boys.

### Statistical analysis

All analyses were conducted in R (version 3.5.0, R Core Team 2018) using RStudio (version 1.1.463, RStudio Team 2016). We used multiple regression to investigate the association between multiple predictor variables and our outcome of interest. We used logistic regression when data were binomially distributed (e.g. 0 or 1 coded data, such as whether or not breastfeeding was initiated), and linear models for normally distributed data. For analyses on breastfeeding and puberty outcome, we first ran a univariable model considering only the explanatory variable in question (e.g. anxiety measures for the breastfeeding model, or breastfeeding measures for subsequent models) and then included all potentially confounding variables. For analyses on growth, our first model also included mass at the start of the time period, and sex. To explore the role of BMI at 10 years as a possible mediator of the effects of breastfeeding on puberty onset, we ran three models for each puberty outcome: one univariate model (association with breastfeeding only), one also including only potential confounds, and a third including potential confounds and BMI at 10 years.

## Results

A summary of all data included in the analyses, including the distribution of both outcome variables of interest and potentially confounding variables is provided in Table 1.

**Table 1.** Summary of data included and tables of variables and confounder variables.

Characteristic	Never breastfed	Breastfed <6 months	Breastfed > =6 months
Aspects of mother:			
*N*	1900	3847	2759
Anxiety based on questionnaire at 32 weeks’ gestation (CCEI range):			
Anxiety 1st quartile (0–2)	482 (25%)	1129 (29%)	841 (30%)
Anxiety 2nd quartile (3–4)	420 (22%)	870 (23%)	713 (26%)
Anxiety 3rd quartile (5–7)	522 (27%)	1031 (27%)	713 (26%)
Anxiety 4th quartile (8–16)	476 (25%)	817 (22%)	492 (18%)
Smoking during pregnancy (yes)	597 (31%)	838 (22%)	341 (12%)
Age at parturition (mean, SD)	27.08 (4.68)	28.49 (4.52)	30.48 (4.20)
Parity (>1 child)	1192 (63%)	1880 (49%)	1622 (59%)
Education (university degree)	53 (3%)	431 (11%)	785 (28%)
BMI (mean, SD)	23.56 (4.39)	23.00 (3.70)	22.35 (3.23)
Traits in girls: (*see below for *N*)			
Growth 0–8 months, kg (mean, SD)	5.23 (0.78)	5.31 (0.88)	4.78 (0.76)
Growth 8–25 months, kg (mean, SD)	4.01 (1.09)	3.91 (0.89)	3.93 (0.79)
Growth 25–61 months, kg (mean, SD)	6.97 (1.77)	7.40 (2.36)	6.90 (1.81)
Age at menarche, years (mean, SD)	12.2 (1.4)	12.2 (1.3)	12.4 (1.3)
BMI at 10 years, kg (mean, SD)	18.80 (3.20)	18.60 (3.26)	17.98 (2.95)
Traits in boys: (*see below for *N*)			
Growth 0–8 months, kg (mean, SD)	5.72 (1.03)	5.77 (0.88)	5.34 (0.93)
Growth 8–25 months, kg (mean, SD)	3.64 (0.91)	3.92 (1.02)	3.97 (0.90)
Growth 25–61 months, kg (mean, SD)	6.64 (1.66)	6.75 (1.74)	6.78 (1.73)
Proportion voice change above median (14.6 years)	49%	52%	52%
BMI at 10 years, kg (mean, SD)	18.11 (3.18)	17.88 (2.70)	18.04 (3.01)

*These data are measured only in a subset of the total sample (*N* = 945, *N* = 828, *N* = 698 for growth 0–8, 8–25 and 25–61, respectively; *N* = 2281 and *N* = 1936 for puberty and BMI in girls and boys, respectively).

### What is the relationship between prenatal anxiety and breastfeeding?

Associations between prenatal anxiety and breastfeeding outcomes are shown in Table 2, both for models that did not include any confounders and that did consider potentially confounding effects of maternal smoking, age, parity, education, and BMI. In univariable analyses, mothers with highest levels of self-reported prenatal anxiety, i.e. in the fourth quartile of the CCEI score, were less likely to ever breastfeed their children (OR [95% CI]: 0.67 [0.58, 0.78]), or to breastfeed beyond six months (0.73 [0.64, 0.83]). These effects were attenuated after adjusting for confounders, particularly for the association between anxiety and breastfeeding duration (Ever breastfed: (0.83 [0.71, 0.97]), Breastfed ≥6 m**:** 0.87 [0.76, 1.01]). The negative association between higher anxiety and breastfeeding onset or duration remained significant when considering anxiety measured on a continuous scale (absolute or quartile terms), or as being above a cut-off value (see Supplementary Appendix).

**Table 2.** Association between anxiety reported by women at 32 weeks’ gestation (CCEI scale in parentheses) and whether they subsequently breastfed their child at all, or breastfed for at least 6 months, both when including no confounders in the model or including all confounders, i.e. smoking, age, parity, education, and BMI.

Outcome variable	Maternal anxiety at 32 wk gestation	Model 1 (no confounders)		Model 2 (all confounders)	
		Odds ratio (95% CI)	*p*-value	Odds ratio (95% CI)	*p*-value
Ever breastfed	1st quartile (0–2)	1 (reference)		1 (reference)	
	2nd quartile (3–4)	0.92 (0.80, 1.07)	0.3	0.96 (0.82, 1.12)	0.6
	3rd quartile (5–7)	0.82 (0.71, 0.94)	0.005	0.9 (0.78, 1.04)	0.2
	4th quartile (8–16)	0.67 (0.58, 0.78)	<0.0001	0.83 (0.71, 0.97)	0.02
Breastfed ≥6m	1st quartile (0–2)	1 (reference)		1 (reference)	
	2nd quartile (3–4)	1.06 (0.94, 1.2)	0.4	1.09 (0.96, 1.25)	0.2
	3rd quartile (5–7)	0.88 (0.78, 0.99)	0.04	0.98 (0.86, 1.11)	0.7
	4th quartile (8–16)	0.73 (0.64, 0.83)	<0.0001	0.87 (0.76, 1.01)	0.06

### What is the association between breastfeeding and later growth and puberty onset?

We categorised breastfed status to include whether or not an individual was breastfed at all, and, if so, whether they were breastfed for less than or more than, or equal to, six months. The associations between these experiences of breastfeeding and growth between 0–8 months, 8–25 months and 25–61 months are provided in Table 3. We found that infants who were breastfed for six months or longer had slower growth than those who were never breastfed or who were breastfed for less than six months (effect of being breastfed ≥6 months [CI]: −0.41 [−0.56, −0.26]), even when confounding variables other than sex and starting mass (included in all models) were taken into account. However, beyond 8 months of age (by which time <33% of babies were receiving breastmilk), there were no clear differences between those who were breastfed for at least six months and those who were not breastfed or breastfed for less than six months (effect of being breastfed ≥6 months [CI] on growth 8–25 months: 0.19 [0.00, 0.38], and on growth 25–61 months: 0.38 [0.00, 0.76]).

**Table 3.** Association between breastfed status (predictor, categorical) and growth (outcome, change in body weight from previous timepoint) at 8 , 25, and 61 months in the Children in Focus subset.

		Outcome: Difference in mass (kg)			
		Model 1 (initial mass + sex)		Model 2 (all confounders)	
Growth period	Breastfed type (predictor)	Beta (95% CI)	*p* Value	Beta (95% CI)	*p* Value
0–8 m	Not breastfed	(reference)		(reference)	
	Breastfed <6 months	0.06 (−0.09, 0.20)	0.5	0.03 (−0.13, 0.18)	0.7
	Breastfed ≥6 months	−0.41 (−0.56, −0.26)	<0.0001	−0.41 (−0.57, −0.24)	<0.0001
8–25 m	Not breastfed	(reference)		(reference)	
	Breastfed <6 months	0.09 (−0.08, 0.26)	0.3	0.06 (−0.11, 0.24)	0.5
	Breastfed ≥6 months	0.16 (−0.02, 0.34)	0.08	0.19 (0.00, 0.38)	0.051
25–61 m	Not breastfed	(reference)		(reference)	
	Breastfed <6 months	0.14 (−0.22, 0.49)	0.5	0.25 (−0.11, 0.61)	0.2
	Breastfed ≥6 months	0.14 (−0.22, 0.50)	0.5	0.38 (0.00, 0.76)	0.05

The baseline model also included mass at the start of the period and sex, whereas the full model included potential confounding covariates of maternal prenatal anxiety, maternal smoking, maternal age, maternal parity, maternal education, and maternal BMI.

Of the confounding variables, only starting mass, sex, parity and maternal BMI explained significant variation in one or all of the models. Notably, there was no additional effect of maternal self-reported anxiety on growth at any of the developmental stages considered (all *p* > 0.34). Moreover, we did not find any independent effect of self-reported anxiety and growth at any stage when we conducted univariable analyses (see Supplementary Appendix).

The associations between breastfed status and puberty onset when potentially confounding or mediating variables were excluded or included in the model are provided in Table 4. We found that girls breastfed for 6 months or more reached menarche slightly later than those who were not breastfed (effect of being breastfed ≥6 months [CI] on age at menarche, in terms of additional months: 2.76 [0.90, 4.63]), but this effect attenuated slightly when confounding variables were included (1.91 [-0.06, 3.88]). Including BMI at 10 years as a potential mediator suggests that the association between breastfeeding and puberty might be, in part, mediated by BMI at 10 years (1.51 [-0.38, 3.40]). In contrast, we found no association between breastfeeding status and whether or not a boy recorded his voice to have changed above the median level of 14.6 years. Moreover, we did not find any independent effect of prenatal anxiety on either age at menarche or age at voice change (see Supplementary Appendix).

**Table 4:** Association between breastfed status and age at onset of puberty, as measured as age at menarche in girls and whether voice first changed above the median in boys.

Outcome: Age at menarche (months)						
Breastfed type	Model 1 (univariable)		Model 2 (confounders*)		Model 3 (conf.+BMI)	
	Beta^a^ (95% CI)	*p* Value	Beta^a^ (95% CI)	*p* Value	Beta^a^ (95% CI)	*p* Value
Not breastfed	(reference)		(reference)		(reference)	
Breastfed <6m	0.57 (−1.32, 2.46)	0.6	0.35 (−1.55, 2.25)	0.7	0.50 (−1.33, 2.33)	0.6
Breastfed ≥6m	2.76 (0.9, 4.63)	0.004	1.91 (−0.06, 3.88)	0.06	1.51 (−0.38, 3.40)	0.1
Outcome: Age at first voice change (above median)						
	Model 1 (univariable)		Model 2 (confounders*)		Model 3 (conf.+BMI)	
Breastfed type	Odds ratio^a^ (95% CI)	*p* Value	Odds ratio^a^ (95% CI)	*p* Value	Odds ratio^a^ (95% CI)	*p* Value
Not breastfed	(reference)		(reference)		(reference)	
Breastfed <6m	1.12 0.87, 1.45)	0.4	1.09 (0.83, 1.42)	0.6	1.08 (0.83, 1.41)	0.5
Breastfed ≥6m	1.12 (0.87, 1.46)	0.4	1.01 (0.76, 1.33)	0.9	1.02 (0.77, 1.34)	0.9

^a^For age at menarche: mean difference in months.

*Potential confounding variables: maternal prenatal anxiety, maternal smoking, maternal age, maternal parity, maternal education, and maternal BMI (model 2). We also considered all variables and a potential mediator of BMI at 10 years (model 3). Note that ‘Effect’ refers to the regression coefficient for the model on age at menarche, and the odds ratio for the model on age at voice change.

For age at voice change: odds ratio between above and below median.

## Discussion

In this study, we investigated associations between prenatal anxiety, breastfeeding and later growth and puberty onset. We found that, as in previous work (Li et al. 2008; Dozier et al. 2012; Ystrom 2012), mothers with higher scores of self-reported anxiety were less likely to breastfeed at all, or for longer than six months. Children who were breastfed for at least six months had lower growth until 8 months but then slightly higher growth until five years of age. Girls who were breastfed for at least six months had later age at menarche, although this association attenuated when considering other confounders, and was potentially mediated through BMI in late childhood. We did not find any independent effect of prenatal anxiety on child growth or age at menarche. Below, we discuss our findings in the context of other work in cohort studies and in light of the evolutionary framework presented in the introduction, while acknowledging the caveats in applying such a framework to a contemporary human population.

The fact that we find a link between prenatal stress, as assessed through self-reported anxiety in late gestation, and the initiation and duration of breastfeeding is not surprising given the extent of evidence for this relationship in other studies (Li et al. 2008; Dozier et al. 2012; Buck et al. 2018). Indeed, higher levels of maternal stress are known to interfere with milk production and the let-down reflex, both through physiological interference in the hormonal pathways involved and through behavioural changes (Dewey 2001). We appreciate, however, that our study is limited in that we rely on self-reported anxiety as direct physiological measures of stress, e.g. circulating cortisol, were not available. Anxiety – in the form of a future-oriented emotional state and apprehensive anticipation of future events – may not necessarily correlate with stress in the sense of the physiological state of an individual when primed to escape from a threat. In related work, we used Mendelian randomisation (Davey Smith and Ebrahim 2003) to investigate associations between single nucleotide polymorphisms associated with cortisol expression and breastfeeding, but the lack of response indicates that associations between anxiety and breastfeeding are likely not the consequence of heightened circulating cortisol levels (India Wright et al., unpublished). A recent systematic review found that studies associating prenatal anxiety with child outcomes are not generally mediated through maternal cortisol levels (Zijlmans et al. 2015), and – in line with these studies – we propose that cortisol is unlikely to be the mechanism underlying the association between anxiety and breastfeeding in our study. In this sense, our study is quite different to those in non-human animals where elevated cortisol is a likely driver of the association between maternal stress and later offspring outcomes (Berghänel et al. 2017).

We then investigated the links between breastfeeding, having found this to be associated with prenatal anxiety, and later-life outcomes including growth and puberty onset. Note that we did not find an independent effect of prenatal anxiety on growth or age at menarche, in contrast to predictions of life history theory described in the introduction (Berghänel et al. 2017). One possibility for this lack of effect is that, as explained above, our measure of self-reported anxiety does not necessarily reflect physiological stress as described in the animal studies. There are other potential reasons: Berghänel et al. (2017) find that the effects of prenatal stress are strongest when experienced early in development, while anxiety here is measured at 32 weeks’ gestation. However, it is unlikely that an earlier measure of anxiety would have shown an effect because, in this cohort study, others have shown that anxiety at 32 weeks' gestation is the most influential for child traits (Nawa et al. 2019), and also correlates with anxiety at 18 weeks' gestation (Heron et al. 2004). More surprisingly, we did not replicate the findings of Nawa et al. (2019) – using the same cohort study – that prenatal anxiety is associated with a higher change in BMI between 25 and 31 months, albeit with confidence intervals close to zero (0.004–0.12). However, both studies have relatively small sample sizes due to the subset of individuals with such detailed growth measures (*n* ∼ 1000 individuals), and we used a different growth measure over a different period (change in body mass at 8–25 months and 25–61 months).

Although we do not find direct effects of prenatal anxiety on later growth or puberty, we do find associations between breastfeeding and these later traits, and breastfeeding is associated with prenatal anxiety. Consequently, our results could be interpreted, albeit tentatively, to fit with some predictions from evolutionary life history theory, that early adversity results in reduced breastfeeding and, in turn, accelerated development in offspring. We emphasise the caveats and challenges in applying such predictions to a human cohort study. We appreciate that there are likely many other unmeasured confounder factor that may shape the observed associations between prenatal anxiety, breastfeeding and later growth and reproductive development. There is great complexity, for example, in whether or not a woman breastfeeds and for how long, affected not only by her levels of anxiety but also societal factors, such as the type of guidance and support she receives about breastfeeding, both in the home and in the community (Dennis 2002). Similarly growth and puberty onset are very complex traits influenced not only by early-life factors but by the current environment as well as genetic effects (e.g., Hollis et al. 2018). It is also important to consider cultural context, as determinants of puberty onset are not consistent across cultures (Sear et al. 2019). We note also whether self-reported anxiety is indeed the most pertinent type of prenatal stressor to consider. While we include maternal smoking as a covariate in all analyses, it may be that prenatal smoking provides a more direct measure of the stress environment *in utero* and its pervasive effects. Indeed, studies in another cohort have shown that girls exposed to maternal smoking have earlier age at menarche (Behie and O’Donnell 2015), and other ALSPAC studies have demonstrated that child growth can even be influenced by their parents’ exposure to grandparental smoking (Golding et al. 2014).

The replacement or supplementation of breastmilk with formula creates an important challenge for applying evolutionary predictions to a human population. In non-human studies in natural populations, a reduction in breastfeeding can be assumed to reflect reduced maternal investment. However, in humans, reduced breastfeeding does not mean less nutrient transfer to young: indeed, the reverse can be the case. Infants reared on formula milk may receive similar, or excess, energy requirements over their breastfed counterparts yet experience different cues of environmental stress or adversity that might be present in breastmilk. While formula milk varies from breastmilk in nutrient composition, great efforts have been made in developing formula which is a close proxy to breastmilk (Martin et al. 2016). However, other dynamic signals based on maternal physiological state might only be directly passed through breastmilk, for example cortisol which is associated with stress (Miller et al. 2013). This potentially allows one to tease apart the nutritional versus non-nutritional signalling inputs such as stress hormones – often difficult in non-human studies – and compare the effects of stress signals from their mother potentially independently of other forms of energetic maternal investment. Another way to overcome the confounding issue of formula feeding would be to examine associations between prenatal stress, anxiety and later-life outcomes among children who were exclusively breastfed. While we did not have the resolution in our dataset to address this question, such an analysis may be better achieved with data from populations with lower rates of formula supplementation.

Finally, we examined the link between breastfeeding outcome and age at puberty onset in girls and boys. We found that, when considering breastfeeding alone, girls who were breastfed for at least six months experienced menarche at a slightly later age, as has been shown in other cohorts (Aghaee et al. 2019). This effect attenuated, however, when body mass index at 10 years was included in the model. Indeed, there is extensive evidence for the association between BMI and puberty onset both in this and other studies (Ong et al. 2009). Thus, the association between breastfed status and puberty may be driven by the link between breastfeeding and later BMI, although this often attenuates when considering other maternal environmental and physiological factors (e.g. in ALSPAC, Toschke et al. 2007, and in studies in general, Owen et al. 2005). Our results thus support the findings by Duchesne et al. (2017) from the Canadian Ice Storm study, that prenatal adversity is associated with earlier age at menarche but mediated through BMI at 5.5 years. Future work on such studies could examine in closer detail the role of breastfeeding in shaping such associations.

We do not find any association between breastfed status and age at onset of puberty in boys, but we acknowledge that our measure for puberty in this study has lower resolution as the exact timing of events was not recorded. Indeed, difficulties in measuring the timing of puberty in boys may be a reason for their underrepresentation in studies on timing of reproduction in humans. Our findings are consistent, however, with a recent meta-analysis on early family conditions and reproductive strategies in human males, which did not find any link between early-life socioeconomic status and timing of puberty onset in males although there were associations with other measures of reproduction (Xu et al. 2018).

Taken together, our work seems to support some, but not all, predictions based on the evolutionary framework for linking prenatal stress and later life history outcomes, through maternal investment in early life (Berghänel et al. 2017), at least for females. Infants exposed to higher stress in early-life – based on maternal anxiety, and also through stress-associated reduction in breastfeeding interaction – have higher growth and then likely higher BMI, and thus have earlier age at onset of puberty. This is similar to the ‘fast’ life history strategy one might predict. Other studies have also shown that adversity experienced early in life can result in accelerated reproductive development in support of these predictions (Nettle 2011). The lack of direct association between prenatal anxiety and child growth or age at menarche in our study means that our measure of maternal stress may not be as pervasive as predicted, and as explained above there are many other confounding variables. In particular, supplementation or replacement of breastmilk with formula feeding is likely an important consideration which does not have a parallel in non-human animal studies (except for farm animals and those reared in captivity), nor would it have been an option for ancestral humans.

Although we described two alternative evolutionary explanations (Figure 1), our empirical data are not able to explicitly support one or the other. Note that to fully test our predictions against life-history theory, we would need to compare outcomes for children when they experience a matched environment to that anticipated in early life and when the environment is mismatched. One might envisage measures that would reflect aspects of environmental adversity, such as occupation, income, house ownership, stressful life events (such as moving house, bereavement and so on) and compare a composite measure of adversity in early life to see how well it reflects that experienced at adulthood. While some but not all of these data are available in our current study, consideration of the pertinent features indicating an adverse environment in the study of children born to the children studied here, i.e. ALSPAC-G2 (Lawlor et al. 2019), would result in a dataset well placed to examine these predictions.

To conclude, we link early-life factors, including maternal anxiety and experience of breastfeeding, to childhood growth and later puberty onset, and attempt to interpret them in light of evolutionary life history theory while at the same time acknowledging the limitations of this approach. We end by briefly mentioning the potential public health implications of these findings. Specifically, improving women’s wellbeing around the time of conception and during pregnancy can lead to higher rates of breastfeeding, as has been suggested in other work. Such an effect not only has consequences for child growth and BMI, but also can have later benefits in delaying the onset of menarche, which is associated with better health outcomes for women (e.g. lower cancer risk, Werneck et al. 2018).

## Supplementary Material

Supplemental Material
