# Supplementary material for: Prenatal anxiety, breastfeeding and child growth and puberty: linking evolutionary models with human cohort studies
Source: Ann Hum Biol. 2020 May 20;47(2):106–15. doi: 10.1080/03014460.2020.1751286 (PMC7261397; doi:10.1080/03014460.2020.1751286)
Supplement: Supplemental Material [file IAHB_A_1751286_SM7951.pdf]

Appendix for: Prenatal anxiety, breastfeeding and child growth and puberty: linking evolutionary models with human cohort studies

Here, we describe additional analysis not included in the main paper.

1. Association between prenatal anxiety (different measures) and breastfeeding

To assess the robustness of our results associating prenatal anxiety with breastfeeding outcome, we repeated the models including anxiety measured in three other ways: as a linear quartile, as a continuous variable, or whether the CCEI measure was 9 or higher ('threshold'). Regardless of the type of anxiety measure used, we found an association with higher levels of anxiety and less propensity to breastfeed at all, or to breastfeed beyond six months (table A1).

Table A1. Association between anxiety reported by women at 32 weeks' gestation (CCEI scale, different measures) and whether they subsequently breastfed their child at all, or breastfed for at least six months, both when including no confounders in the model or including all confounders, i.e. smoking, age, parity, education, and BMI. Note that each row represents a different model.

Outcome: Ever breastfed

	Model 1 (no confounders)		Model 2 (all confounders)	
**Anxiety term in model**	**Odds ratio (95% CI)**	**P-value**	**Odds ratio (95% CI)**	**P-value**
CCEI score quartile (linear)	0.88 (0.84, 0.92)	<0.0001	0.94 (0.90, 0.99)	0.01
CCEI score continuous	0.96 (0.94, 0.97)	<0.0001	0.98 (0.96, 0.99)	0.006
CCEI score threshold	0.70 (0.61, 0.80)	<0.0001	0.83 (0.72, 0.96)	0.01

Outcome: Breastfed >6 months

	Model 1 (no confounders)		Model 2 (all confounders*)	
**Anxiety term in model**	**Odds ratio (95% CI)**	**P-value**	**Odds ratio (95% CI)**	**P-value**
CCEI score quartile (linear)	0.90 (0.86, 0.94)	<0.0001	0.96 (0.91, 1.00)	0.05
CCEI score continuous	0.96 (0.95, 0.97)	<0.0001	0.98 (0.96, 0.99)	0.002
CCEI score threshold	0.75 (0.66, 0.85)	<0.0001	0.86 (0.75, 0.99)	0.03

For ease of presenting effect sizes, in the following tables we present associations between anxiety as a threshold measure (CCEI score of 9 or above), but we note that the statistical trends are the same for all other measures of anxiety considered.

2. Association between prenatal anxiety and growth at different stages

We examined whether there was an independent association between prenatal anxiety and child growth at any of the growth stages considered (0–8, 8–25 and 25–61 months) but we did not find any effect, when considering baseline confounders (initial mass and child sex) or all other confounders (table A2).

Table A2. Association between prenatal anxiety (predictor, values shown for CCEI 9 or above compared) and growth (outcome, change in body weight from previous timepoint) at 8 months, 25 months and 61 months in the Children in Focus subset. The baseline model also included mass at the start of the period and sex, whereas the full model included potential confounding covariates of breastfeeding experience, maternal smoking, maternal age, maternal parity, maternal education, and maternal BMI.

	Outcome: Change in mass (kg) Predictor: prenatal anxiety (threshold)			
**Growth stage**	**Model 1 (mass + sex)**		**Model 2 (all confounders)**	
	**Beta (95% CI)**	**P-value**	**Beta (95% CI)**	**P-value**
**0-8 months**	0.10 (-0.06, 0.25)	0.2	0.11 (-0.04, 0.27)	0.2
**8-25 months**	-0.03 (-0.21, 0.15)	0.8	0.02 (-0.16, 0.21)	0.8
**25-61 months**	0.02 (-0.36, 0.39)	0.9	0.05 (-0.32, 0.43)	0.8

3. Association between prenatal anxiety and age at onset of puberty

We examined whether there was an independent association between prenatal anxiety and age at the onset of puberty in girls or boys, but we did not find any effect (table A3).

Table A3. Association between prenatal anxiety and age at onset of puberty (age at menarche in girls, whether voice first changed above the median in boys). Confounding variables: breastfeeding experience, maternal smoking, maternal age, maternal parity, maternal education, and maternal BMI, as well as the potential mediator of BMI at 10 years. Note that 'Effect' refers to the regression coefficient for the model on age at menarche, and the odds ratio for the model on age at voice change.

	**Model 1 (univariable)**		**Model 2 (confounders +BMI)**	
**Predictor**	**Effect[a] (95% CI)**	**P-value**	**Effect[a] (95% CI)**	**P-value**
	**Age at menarche (months)**			
Prenatal anxiety	-0.87 (-2.78, 1.05)	0.4	-0.56 (-2.4, 1.27)	0.5
	**Age at voice change (above median)**			
Prenatal anxiety	0.77 (0.6, 1.00)	0.05	0.81 (0.62, 1.06)	0.1

[a] **For age at menarche: mean difference in months**
For age at voice change: odds ratio between above and below median
